# Release of Doxorubicin by a Folate-Grafted, Chitosan-Coated Magnetic Nanoparticle

**DOI:** 10.3390/nano7040085

**Published:** 2017-04-13

**Authors:** Chung-Lin Yang, Jyh-Ping Chen, Kuo-Chen Wei, Ju-Yu Chen, Chia-Wen Huang, Zi-Xian Liao

**Affiliations:** 1 Department of Neurosurgery, Chang Gung Memorial Hospital, Taoyuan 33305, Taiwan; a0955498306@gmail.com (C.-L.Y.); kuochenwei@adm.cgmh.org.tw (K.-C.W.); santuryqq@gmail.com (J.-Y.C.); chiawen0712@gmail.com (C.-W.H.); 2 Department of Chemical and Materials Engineering, Chang Gung University, Taoyuan 33305, Taiwan; jpchen@mail.cgu.edu.tw; 3 Institute of Medical Science and Technology, National Sun Yat-sen University, Kaohsiung 80424, Taiwan

**Keywords:** magnetic nanoparticles, doxorubicin, chitosan, remote delivery, antitumor effect

## Abstract

In clinical tumor therapy, chemotherapeutic routes have caused severe side effects; current delivery methods are unsatisfactory. Successful design of a remotely folate (FA)-grafted chitosan (CS)-coated magnetic nanoparticle (MNP) with low toxicity, has been achieved. A chemotherapeutic drug such as doxorubicin (DOX), is loaded in the MNP-based matrix (FA-grafted CS-DOX-_TPP_-MNP), which is coated by an activated target tumor molecule of FA-grafted CS biopolymer with the inclusion of tripolyphosphate (TPP) as a linker. The resultant nano-complexes exhibited random aggregates (~240 nm) and zeta potential (−24.9 mV). In vivo experiments using athymic BALB/c nude mice with human glioblastoma U87 cells in a subcutaneous tumor model revealed that magnetic guidance of FA-grafted CS-DOX-_TPP_-MNP, injected via the tail vein, significantly decreased tumor growth. This manuscript demonstrates the feasibility of magnetizing control of FA-grafted CS-DOX-_TPP_-MNP to enhance the localization of drug release.

## 1. Introduction

Chemotherapeutic agents have been shown to be effective against in vivo solid tumors [1,2,3]. Poor clinical outcomes and off-target cytotoxicity are presumably the results of challenges inherent in tumor-drug delivery via systemic administration [4]. Among the various chemotherapeutic agents, doxorubicin (DOX) is a type of chemotherapy drug that is also known as an anthracycline. Furthermore, DOX slows or stops the growth of cancer cells, and its mechanism is associated with the inhabitation of topoisomerase II, which cancer cells need to divide and grow [5]. However, a number of improvements are sought in order to more broadly develop chemotherapy. A particular challenge is how to systemically administer chemotherapy and overcome limitations in off-targets, as well as side effects such as cardiotoxicity and drug resistance—especially for tumors within sensitive tissues/organs.

Among polymeric or metallic nano-carriers, the tumor microenvironment has highly-activated pH-sensitive polymeric carriers and triggers drug/protein release from the carrier [6,7,8]. However, the characteristics of the tumor microenvironment are dependent on in vivo solid tumor size or volume. Specifically, magnetic nanoparticles (MNP) provide accelerated MNP accumulation in targets when directed by magnetic-field-enforced distribution, due to desirable magnetic properties and low toxicity [9]. Furthermore, MNPs with high magnetic relaxivities can be easily dispersed in various aqueous solutions through the ligand exchange method [10]. Over the past several years, effective magnetically-mediated delivery technologies have shown significant potential in biomedical applications [9,10,11], and have inspired various approaches to promote delivery to specific sites [9]. For example, DNA vaccines using MNP-based nanoparticles offer improved delivery, and can induce a significant immune response in mouse studies [12]. Most clinical trials using virotherapy have treated via intratumoral injection [13]; however, a significant technique needed for clinical virotherapy is improved systemic delivery [14]. As an alternative treatment, we have successfully designed a combined hybrid system of virus and MNPs, using chemical conjugation for controllable micro-virotherapy under magnetic fields [9].

**Figure 1:**
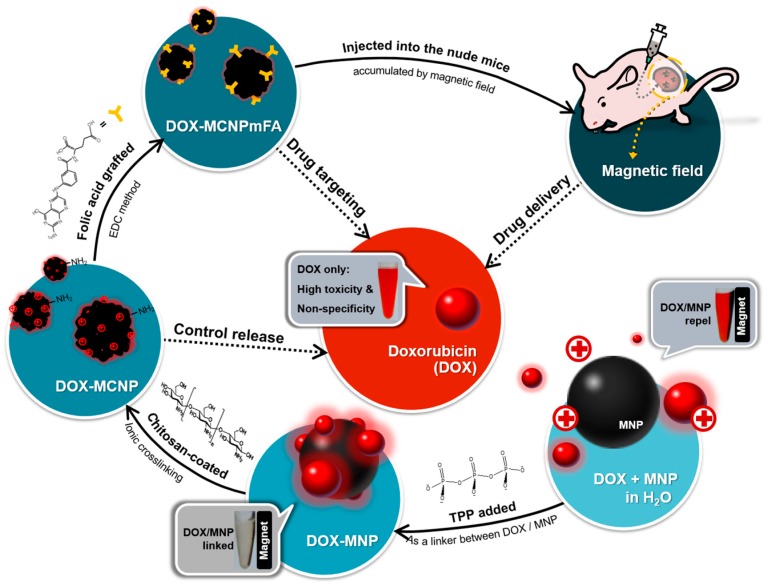
Conceptual scheme of the approach for multitasking drug magnetic carriers. DOX: doxorubicin; EDC: 1-Ethyl-3-(3-dimethylaminopropyl)-carbodiimide; MNP: magnetic nanoparticles; TPP: tripolyphosphate; FA: folic acid; MCNP: CS-_TPP_-MNP; MCNPmFA: FA-grafted CS-_TPP_-MNP.

Chitosan (CS) is a cationic polysaccharide with low toxicity, and has subsequently been considered for the delivery of DOX [15], antibody [16], or small interfering RNA (siRNA) [17]. CS is also increasingly used for various biomedical applications, including tissue engineering [18], wound healing [19], and antimicrobial uses [20]. Alternately, the folate (FA) receptor is a highly-overexpressed receptor on many cancer cell line surfaces [21,22]. Moreover, FA grafted or coated nanoparticles have exhibited a considerable enhancement of cellular uptake compared with un-modified nanoparticles [23,24]. Taken together, this provides solid motivation for the design of a remotely-directed and highly-targeted tumor delivery of a combination of FA- and CS-coated, DOX-based MNPs for micro-chemotherapy. Use of FA-grafted nanoparticles [25] or CS nanocarriers [26] have exhibited no visible hemolysis effects. An established concept would provide well-defined micro-chemotherapy (Figure 1). The validity of this concept was tested with an FA-grafted, CS-coated approach to DOX-based MNPs through the ionic crosslinker tripolyphosphate (TPP), circumventing DOX-based side effects and providing a highly-specific tumor for micro-chemotherapy.

## 2. Results and Discussion

### 2.1. Characterization of Synthesized Particles

**Figure 2:**
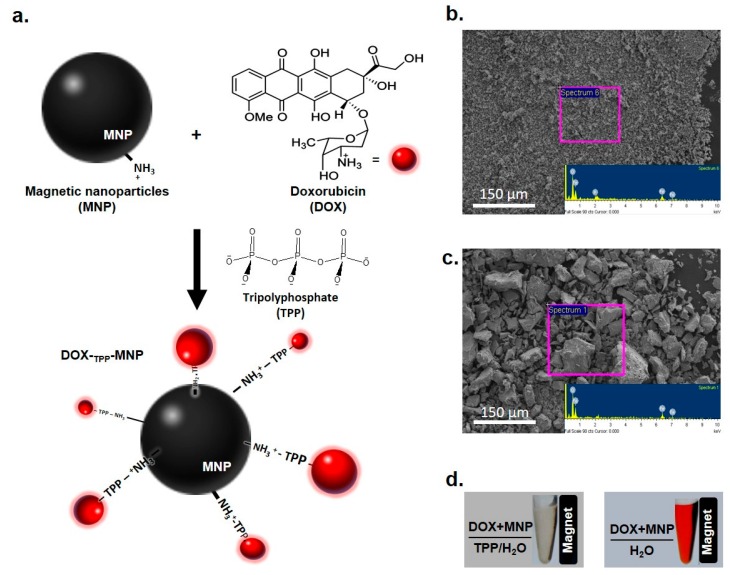
Doxorubicin (DOX) encapsulation with magnetic nanoparticle (MNP) through a linker of tripolyphosphate (TPP). (**a**) Synthesis of DOX-_TPP_-MNP via complexation of the MNP with DOX and TPP. Morphology of (**b**) DOX-_TPP_-MNP or (**c**) DOX-MNP using scanning electron microscopy (SEM) at 200× magnification, and energy-dispersive X-ray (EDAX) analysis of the energy profiles of DOX-_TPP_-MNP or DOX-MNP. (**d**) Photograph of DOX-_TPP_-MNP and DOX-MNP in a 1.5-mL microcentrifuge tube; a magnet was applied to the right side of microcentrifuge tube.

MNP formation using chemical coprecipitation was carried out using amino groups on the MNP surface [27], and these amino groups led to a positive charge (+37.4 ± 1.74 mV) at pH < 7 (Table 1). Furthermore, MNPs were bound to DOX to form a DOX-_TPP_-MNP (zeta potential: +21.30 ± 2.69 mV) using TPP as a negatively-charged linker (Figure 2a). SEM morphology of DOX-_TPP_-MNP displayed a uniform surface, and the energy-dispersive X-ray (EDAX) analysis spectrum indicated that P, Fe, and O were contributed by DOX-_TPP_-MNP (Figure 2b). In contrast, the SEM image of DOX-MNP formation without TPP addition, showed asymmetrical particles, and the EDAX analysis spectrum indicated Fe and O (Figure 2c). Alternately, DOX-_TPP_-MNP showed a clear aqueous solution when a magnetic field was applied on the right side in a 1.5-mL microcentrifuge tube (Figure 2d). DOX-NMPs displayed a clear red supernatant, separated from the magnetic nanoparticles, after the magnetic field was used in the 1.5-mL microcentrifuge tube. These results clearly indicate that TPP acts as a crosslinker between ionic crosslinks of tertiary amines of DOX and CS [28].

**Figure 3:**
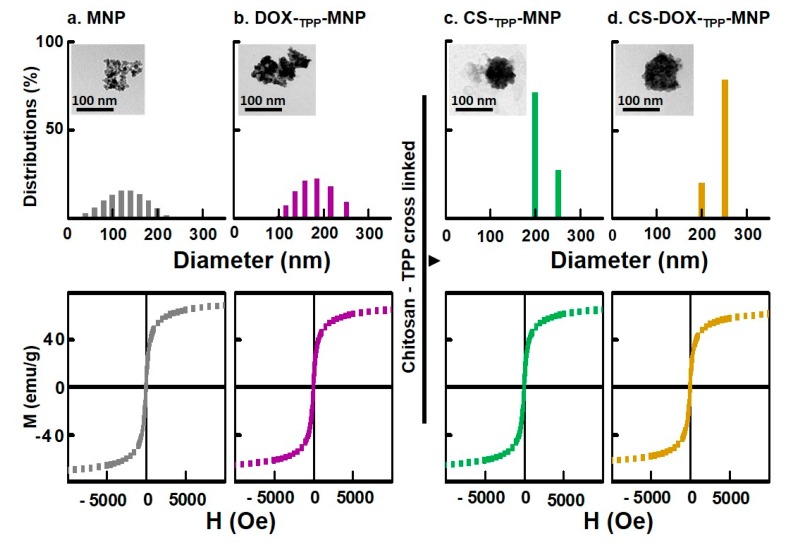
Characterizations of the synthesized nanoparticles. Size distribution and magnetization curves of (**a**) MNP, (**b**) DOX-_TPP_-MNP, (**c**) CS-_TPP_-MNP, and (**d**) CS-DOX-_TPP_-MNP. Inset is the TEM image of the nanoparticles. Bars = 100 nm.

Among the various synthesized nanoparticles, TEM images and particle size measurements illustrated MNP or DOX-_TPP_-MNP aggregation, and consequently produced a wide distribution of particle sizes (Figure 3a,b). In contrast, the size distribution of CS-_TPP_-MNP or CS-DOX-_TPP_-MNP had a uniform size distribution when CS was added to the DOX-_TPP_-MNP system (Figure 3c,d). Likewise, TEM morphology of CS-_TPP_-MNP or CS-DOX-_TPP_-MNP showed a spherical particle. Additionally, the representative hysteresis loops of the synthesized nanoparticles at an ambient temperature had a saturation magnetization of 57.5–68.7 emu·g^−1^, since the MNPs had ca. 68.7 emu·g^−1^ (Table 1). The saturation magnetization of various synthesized MNPs were mostly attributed to the existence of the coated materials on the surfaces of the MNPs. Overall, neither CS coating nor ionic crosslinking of TPP affected the MNP’s unique magnetic property.

### 2.2. Profile of DOX Release from DOX-_TPP_-MNP or CS-DOX-_TPP_-MNP

**Figure 4:**
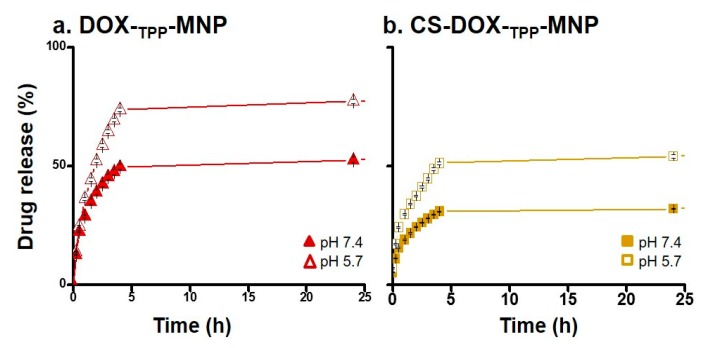
DOX release prolife of synthesized nanoparticles at different pH values. Release rate of DOX from (**a**) DOX-_TPP_-MNP and (**b**) CS-DOX-_TPP_-MNP in phosphate-buffered saline (PBS) solution at pH 7.4 or pH 5.6, with different incubation periods. Results show mean of measurements conducted in triplicate, standard deviation (±s.d.).

To mimic DOX release from DOX-_TPP_-MNP or CS-DOX-_TPP_-MNP in the bloodstream and the endosome/lysosome, the in vitro profiles of DOX release in phosphate-buffered saline (PBS) solution at pH 7.4 or pH 5.7 for difference incubation times, were evaluated. The release prolife of DOX from DOX-_TPP_-MNP exhibited a burst release due to CS uncoated with DOX-_TPP_-MNP when incubated at pHs of 7.4 or 5.7 (Figure 4a). Similarly, released DOX profiles of DOX-_TPP_-MNP clearly showed a burst release of DOX at pH 5.7. Taken together, these results suggest that DOX-_TPP_-MNP had an unstable status in neutral or acidic solutions. In contrast, the released DOX profiles of CS-DOX-_TPP_-MNP displayed a stable release of DOX (Figure 4b). Overall, the total amount of DOX released from DOX-_TPP_-MNP or CS-DOX-_TPP_-MNP at pH 7.4 was 52.55% or 31.97%, 24 h post-incubation. Taken together, these results clearly indicate that CS-DOX-_TPP_-MNP exhibited a gradual DOX release from nano-carriers after the CS coating process.

### 2.3. Effect of FA Molecules on Size Distribution of Synthesized Nanoparticles

**Figure 5:**
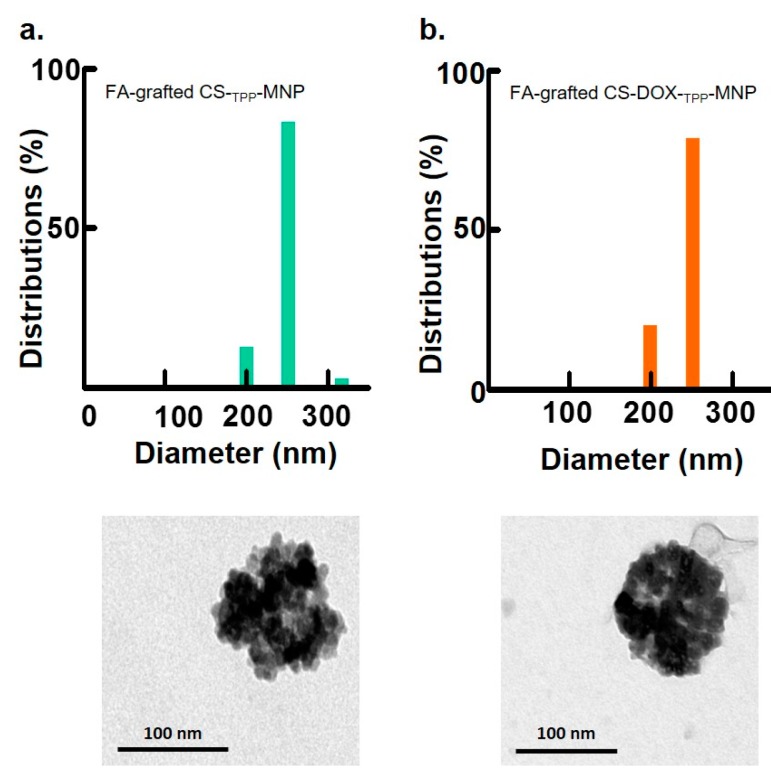
Characterization of synthesized nanoparticles. Size distribution and magnetization curves of (**a**) FA-grafted CS-_TPP_-MNP or (**b**) FA-grafted CS-DOX-_TPP_-MNP. Inset is a TEM image of the nanoparticles. Bars = 100 nm.

DOX is an anthracycline chemotherapeutic drug that causes apoptotic cell death, particularly in exponentially-growing cells. However, the occurrence of side-effects is considerable when patients received chemotherapeutic treatment using DOX [4]. A particular challenge is how to administer DOX systemically and also how to overcome off targeting limitations. Commonly, the folate (FA) receptor is a highly-overexpressed receptor on the surface of many cancer cell lines [21,22,23,24,29]. To improve the selection of DOX for a tumor and to decrease the side-effects associated with DOX release, FA was grafted to CS via 1-ethyl-3-(3-dimethylaminopropyl)-carbodiimide (EDC)/*N*-hydroxysuccinimide (NHS) conjugation, as was done in previous studies [9]. The results show that FA-grafted CS-_TPP_-MNP without the addition of DOX had a stable distribution of particle sizes (Figure 5a). After FA-grafted CS was coated on DOX-_TPP_-MNP, the FA-grafted CS-DOX-_TPP_-MNP still had a uniform size distribution (Figure 5b), with a hydrodynamic diameter of 246.8 ± 5.52 nm, as illustrated in Table 1. Furthermore, FA-grafted CS-DOX-_TPP_-MNP had a hydrodynamic diameter of ca. 270–290 nm in a culture medium (Table 1). However, FA-grafted CS-DOX-_TPP_-MNP’s zeta potential was converted to −24.9 ± 0.66 from +31.93 ± 0.96 mV (CS-DOX-_TPP_-MNP), due to a negative FA-grafted CS-coated layer.

### 2.4. In Vitro Micro-Chemotherapy and Cytotoxicity of Synthesized Nanoparticles

**Figure 6:**
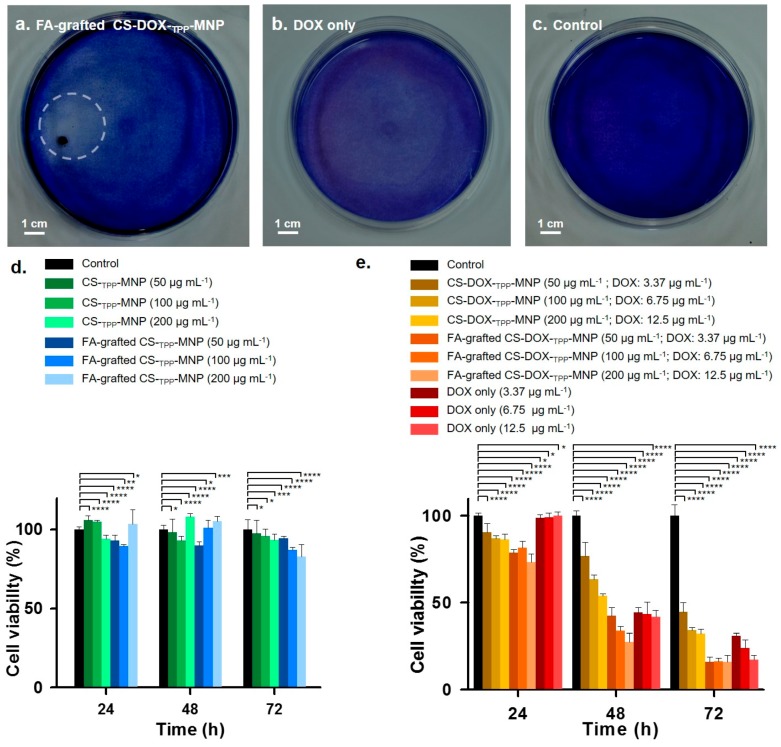
Localized DOX release from synthesized nanoparticles and cytotoxicity. Hematoxylin-stained photographs of localized DOX delivery using (**a**) FA-grafted CS-DOX-_TPP_-MNP, (**b**) only DOX, or (**c**) phosphate-buffered saline (PBS). Human glioblastoma U87 cells were incubated with nanoparticles for 72 h with stirring at 60 rpm in culture medium at pH 7.4, and fixed on the circular magnet at the back of the culture plate. After 72 h, the treated cells were incubated for 10 min using a hematoxylin-stained assay. Cell viability after treatment with (**d**) CS-_TPP_-MNP or FA-grafted CS-_TPP_-MNP at various concentrations and (**e**) CS-DOX-_TPP_-MNP, FA-grafted CS-DOX-_TPP_-MNP, or only DOX. The viability of untreated U87 cells was assigned a value of 100% and measured using an MTT assay (* *p* > 0.2, ** *p* = 0.1, *** *p* < 0.1, **** *p* < 0.005; based on a two-tailed *t*-test, assuming unequal variances). Results show the mean of measurements conducted in triplicate ± s.d.

To examine the remotely-controlled synthesized nanoparticles for localized DOX delivery, human glioblastoma U87 cells were incubated with FA-grafted CS-DOX-_TPP_-MNP or only DOX for 72 h of incubation, and culture dishes were stirred at 60 rpm. After 72 h of treatment, live cells were stained using a hematoxylin-stained assay for 10 min. In the FA-grafted CS-DOX-_TPP_-MNP treatment, appreciable blue-based colorless area accumulated with a magnetic field (1400 Gauss) to produce a micro-sized anti-tumor proliferation of U87 cells, which only resulted in a circular area (Figure 6a). In contrast, unmodified DOX had a homogenous and random cell-death distribution compared with the negative control (Figure 6b,c). This result suggests that FA-grafted CS-DOX-_TPP_-MNP may have a promising effect on the off-target of chemotherapy.

In general, the nano-carrier used in chemotherapeutic drugs or proteins must be low in cytotoxicity [6,7,8]. To evaluate the cytotoxicity of U87 cells treated with CS-_TPP_-MNP, CS-DOX-_TPP_-MNP FA-grafted CS-_TPP_-MNP, or FA-grafted CS-DOX-_TPP_-MNP at various concentrations, for 24, 48, or 72 h incubation, cells viability was determined using an (3-(4,5-Dimethylthiazol-2-yl)-2,5-Diphenyltetrazolium Bromide) (MTT) assay. No cytotoxicity was observed for CS-_TPP_-MNP or FA-grafted CS-_TPP_-MNP incubated with cells, representing a lack of any serious factors such as FA-grafted, CS carrier, a crosslinker of TPP, or MNPs (Figure 6d). Impressively, FA-grafted CS-DOX-_TPP_-MNP at various concentrations exhibited significant cytotoxicity compared with CS-DOX-_TPP_-MNP or only DOX when incubated for 24, 48, or 72 h (Figure 6e). These results clearly indicate that FA-grafted CS-DOX-_TPP_-MNP may directly bind with U87 cells through FA ligand–receptor interaction and subsequent release of DOX from the carrier in the cells. Overall, FA-grafted CS-DOX-_TPP_-MNP was suited to efficient and highly-specific tumor delivery.

### 2.5. In Vivo Antitumor Effect

**Figure 7:**
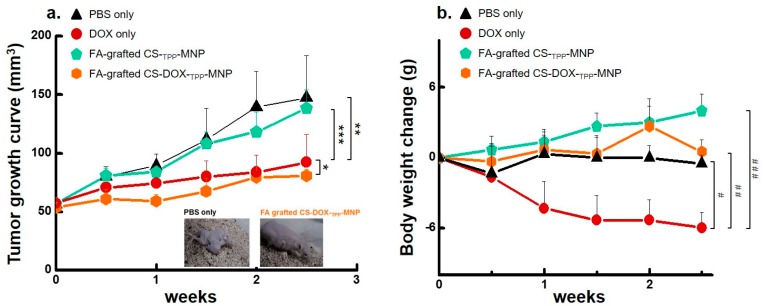
In vivo antitumor therapy under various conditions. (**a**) Effect of magnetic-targeting nanoparticles on tumor volumes via intravenous injection. Athymic BALB/c nude mice with a human glioblastoma U87 cells subcutaneous tumor were injected using various treatments. Tumor sizes were measured using a caliper on the described days (* *p* = 0.78; ** *p* > 0.09; *** *p* < 0.05; based on a two-tailed *t*-test, assuming unequal variances). Results show the mean of measurements conducted in triplicate ± s.d. Representative images of treated mice, two weeks post-treatment. (**b**) Body weight of mice over time in response to treatments using various nanoparticle formulations via intravenous injection (^#^ *p* = 0.23; ^##^ *p* < 0.01; ^###^ *p* < 0.0015; based on a two-tailed *t* test, assuming unequal variances). Results show mean of measurements conducted in triplicate ± s.d.

To improve antitumor therapeutic effects and to decrease the side effects of doxorubicin (DOX) through the bloodstream, we developed a nano-carrier of FA-grafted CS-_TPP_-MNP with a low cytotoxicity to encapsulate with DOX (Figure 1, Figure 2, Figure 3, Figure 4, Figure 5 and Figure 6). Furthermore, FA-grafted CS nanocarriers have negligible hemolytic activity [26]. To validate these data in vivo, we administered carriers to an athymic BALB/c nude mouse with an aggressive subcutaneous U87 cells tumor via tail-vein injection and analyzed the measurements of tumor volume and mouse body weight. All tumors from mice were injected via the tail vein with formulations containing FA-grafted CS-_TPP_-MNP, FA-grafted CS-DOX-_TPP_-MNP, only DOX, or PBS, and all tumors were exposed to magnetic fields for one hour. Remarkable tumor suppression was achieved when the FA-grafted CS-DOX-_TPP_-MNP or only DOX were systemically administered via a tail vein injection (*p* < 0.02, Figure 7a). These data imply a successful remote delivery of FA-grafted CS-DOX-_TPP_-MNPs to the tumor site via cellular uptake and a localized and highly-efficient release of DOX. No tumor growth suppression was observed when PBS or FA-grafted CS-_TPP_-MNPs were administered via tail-vein injection. Body weights remained stable over the FA-grafted CS-DOX-_TPP_-MNP treatment periods, and were similar to those of the controls of FA-grafted CS-_TPP_-MNP or PBS (Figure 7b). Moreover, DOX treatment led to a significant decrease in body weight compared with PBS (*p* < 0.0015). Taken together, our designed FA-grafted CS-DOX-_TPP_-MNP system achieved tumor suppression through localized release of DOX via a tail-vein injection.

## 3. Materials and Methods

### 3.1. Materials

Chitosan (CS, low viscosity, *M*w = 50–190 kDa) and ferric chloride 4-hydrate (FeCl_2_·4H_2_O) were obtained from Fluka (Buchs, Switzerland). Doxorubicin hydrochloride (DOX), folic acid (FA), hematoxylin, and sodium tripolyphosphate (TPP) were obtained from Sigma-Aldrich (St. Louis, MO, USA). 1-Ethyl-3-3-dimethylaminopropyl carbodiimide (EDC) and *N*-hydroxysuccinimide (NHS) were obtained from Acros (Pittsburgh, PA, USA). Ferric chloride 6-hydrate (FeCl_3_·6H_2_O) was purchased from J.T. Baker (Philipsburg, NJ, USA).

### 3.2. Synthesis and Preparation of MNP, DOX-_TPP_-MNP, CS-DOX-_TPP_-MNP, or FA-Grafted CS-DOX-_TPP_-MNP

MNPs (magnetic nanoparticles) were synthesized using the chemical co-precipitation method of Molday [21]. A mixture solution of FeCl_3_ and FeSO_4_ (molar ratio 2:1) was heated to 80 °C with deaeration of O_2_ by N_2_ shielding. Subsequently, 10 mL of aqueous ammonia solution was poured into the solution under vigorous stirring. A black precipitate was formed and was washed several times with deionized (D.I.) water. The collected MNP solution was freeze-dried for 24 h.

Thirty milligrams of MNP powder were added to TPP (2.72 × 10^−9^ mole) in 1 mL of 0.3% acetic acid solution (*v*/*v*). The _TPP_-MNP solution was then obtained using sonication for 1 h at 4 °C. Then, 50 μL of DOX solution (6 mg·mL^−1^) was dropped into 350 μL of _TPP_-MNP solution (Figure 2a). DOX-_TPP_-MNPs were formed by sonication for 30 min at 4 °C.

CS-DOX-_TPP_-MNP or FA-grafted CS-DOX-_TPP_-MNP were prepared by dropping either 0.3% CS or 0.3% FA-grafted CS22 with DOX-_TPP_-MNP solution at a ratio of 1:4, under sonication at 4 °C for 1 h. In the control, CS-_TPP_-MNP was prepared by adding 100 μL of CS solution to 400 μL of _TPP_-MNP solution (30 mg·mL^−1^) under sonication at 4 °C for 1 h. FA-grafted CS-_TPP_-MNP was obtained by adding 100 μL of 0.3% FA-grafted CS solution into 400 μL of _TPP_-MNP solution (30 mg·mL^−1^) drop-by-drop, under sonication at 4 °C for 1 h. To remove the unreacted FA-grafted CS or CS, all the synthesized nanoparticles were purified using a magnetic field with repetitive cycles of washing with D.I. water. These final products were stored in a refrigerator at 4 °C before being subjected to further analyses and experiments.

### 3.3. Characterizations

Magnetic characterization measurements were carried out using a Superconducting Quantum Interference Device (SQUID, MPMS XL-7, Quantum Design Inc., San Diego, CA, USA). Particle morphology and the size of the samples in deionized water or culture medium were determined using a scanning electron microscope (SEM, Hitachi, S-3000N, Los Angeles, CA, USA) and dynamic light scattering (DLS, Malvern, Worcestershire, UK), respectively.

A drop of the nanoparticle solution was allowed to air-dry onto a Formvar-carbon-coated 200 mesh copper grid for transmission electronic microscopy (TEM, JEOL, JEM-200 EII, Boston, MA, USA). TEM images were then acquired using a JEOL-1010 microscope, operating at an accelerating voltage of 100 kV.

### 3.4. Encapsulation Efficiency and DOX Release of DOX-_TPP_-MNP, CS-DOX-_TPP_-MNP, or FA-Grafted CS-DOX-_TPP_-MNP

The encapsulated DOX was completely released from DOX-_TPP_-MNP, CS-DOX-_TPP_-MNP, or FA-grafted CS-DOX-_TPP_-MNP by the addition of glacial acetic acid. The encapsulation efficiency of DOX was determined using the following formula: E.E. (%) = ([DOX]_total amount_ − [DOX]_free amount_)/[DOX]_total amount_ × 100%. The E.E. of DOX-_TPP_-MNP, CS-DOX-_TPP_-MNP, or FA-grafted CS-DOX-_TPP_-MNP were 98.20 ± 0.5, 91.56 ± 0.23, and 98.61 ± 0.16, respectively.

The stabilities of DOX-_TPP_-MNP, CS-DOX-_TPP_-MNP, or FA-grafted CS-DOX-_TPP_-MNP were determined by the fractional release of DOX when incubated for various time intervals. DOX-_TPP_-MNP, CS-DOX-_TPP_-MNP, or FA-grafted CS-DOX-_TPP_-MNP were dissolved in 0.5 mL PBS (pH 7.5 and pH 5.7) at 37 °C in a water bath and stirred at 100 rpm. Released DOX in the supernatant was collected when DOX was released from DOX-_TPP_-MNP, CS-DOX-_TPP_-MNP, or FA-grafted CS-DOX-_TPP_-MNP, and the amounts were measured to evaluate the stability of the synthesized nanoparticles. The DOX absorbance at 496 nm was measured by using a UV spectrophotometer (ELISA Reader, Tecan-Sunrise, Männedorf, Switzerland)).

### 3.5. In Vitro Anti-Tumor Proliferation and Cytotoxicity of Synthesized Nanoparticles

The localized released of DOX was performed on human glioblastoma U87 cells grown in 100-mm culture dishes with an attached magnet (diameter: 5 mm, 1400 Gauss). Cells were maintained in DMEM medium supplemented with 10% fetal bovine serum (FBS) and NaHCO_3_ at 37 °C in a humidified incubator. Human glioblastoma U87 cells were incubated with FA-grafted CS-DOX-_TPP_-MNP (concentration: 200 μg·mL^−1^; DOX: 12.5 μg·mL^−1^) or only DOX (concertation: 12.5 μg·mL^−1^), which were premixed in DMEM medium for 72 h under constant stirring at 60 rpm. Localized drug release observations were expressed by number of live cells stained with hematoxylin for 10 min.

The cytotoxicity of the synthesized nanoparticles and DOX only were determined using the MTT (3-(4,5-cimethylthiazol-2-yl)-2,5-diphenyl tetrazolium bromide) method for the human glioblastoma U87 cell line. Briefly, 2.5 × 10^5^ cells were seeded in 96-well plates and incubated for 24 h to allow the cells to attach. The cells were exposed to the synthesized nanoparticles or only DOX at various concentrations (200 μg·mL^−1^/DOX: 12.5 μg·mL^−1^; 100 μg·mL^−1^/DOX: 6.75 μg·mL^−1^; 50 μg·mL^−1^/DOX: 3.37 μg·mL^−1^) at 37 °C for 24, 48, or 72 h. Cell viability was expressed as survival percentage compared with controls where the cells were not exposed to any chemicals, and was set at 100%.

### 3.6. Mouse Studies

All procedures involving animals were permitted by the Laboratory Animal Center of Chang Gung University. Experiments were performed after inoculation of athymic BALB/c nude mice (6 weeks-old male, National Laboratory Animal Center, Taiwan) with human glioblastoma U87 cells (100 μL PBS, 2 × 10^6^ cells). Mice were maintained in a controlled environment with a 12 h/12 h light/dark cycle, and were housed in groups with a maximum of five mice and were allowed food and water ad libitum.

Animals were randomized and divided into groups, with three mice per group for PBS only, DOX only (5 mg·kg^−1^ body weight), FA-grafted CS-_TPP_-MNP, or FA-grafted CS-DOX-_TPP_-MNP (5 mg·kg^−1^ body weight). Once the tumors reached a volume of ~200 mm^3^, the mice were injected with 100 μL of PBS with the test samples via intravenous, two times a week. In the treatments where a magnetic field was applied to the targeted tumor, the U87 xenograft tumors were exposed to magnetic fields of 1400 Gauss for 1 h. Tumor volume was measured using calipers, and the length (*L*) and width (*W*) of the tumor was measured; the tumor volume was calculated according to the following formula: Tumor volume = (0.5 *L*^2^) *W*. Tumor size examination was conducted 24 h after the last treatment. Body weight and tumor volume were measured on alternate days for 3 weeks post treatment. Treatment with PBS injections was used as control. The mice were sacrificed when the tumor volume reached nearly 2000 mm^3^.

### 3.7. Statistical Analyses

Data are shown as mean ± the standard deviation for experiments, performed in triplicate (in vitro and in vivo studies). For statistical significance testing, *p*-values were calculated using a two-tailed *t*-test, assuming unequal variances.

## 4. Conclusions

Doxorubicin encapsulation with folate (FA)-grafted chitosan (CS)-coated magnetic nanoparticles (MNP) with the potential for efficient and micro-sized drug release was developed. FA-grafted CS-DOX-_TPP_-MNP achieved efficient antitumor proliferation in solid tumors from systemic circulation under magnetic fields. Furthermore, in vivo experiments revealed that magnetic guidance of FA-grafted CS-DOX-_TPP_-MNP, injected via the tail vein, significantly improved antitumor effects. No significant loss of body weight was detected, representing a lack of any serious FA-grafted CS-DOX-_TPP_-MNP or magnetic field exposure. Overall, the future development of this material could involve loading both hydrophobic and hydrophilic chemotherapeutic drugs for dual/multiple delivery applications. Such a flexible modality adds greater potential in overcoming inter-individual variabilities in therapeutic responses with improved specificities.

## Figures and Tables

**Table 1:** Parameters of zeta potential, hydrodynamic size, and saturation magnetization of the synthesized nanoparticles. CS: chitosan; FA: folate.

	MNP	DOX-_TPP_-MNP	CS-DOX-_TPP_-MNP	FA-Grafted CS-DOX-_TPP_-MNP
Zeta (mV)	+37.4 ±1.74	+21.30 ± 2.69	+31.93 ± 0.96	−24.9 ± 0.66
Size (nm)	133.0 ± 8.9	180.2 ± 0.92	241.6 ± 1.82	246.8 ± 5.52 (D.I. water) 281 ± 10.31 (Culture medium)
Ms (emu·g^−1^)	68.7	67.8	62.0	57.5
Mr (emu·g^−1^)	1.23	1.54	1.74	1.38
